# Supplementary material for: Are carbon emissions trading and green financial instruments synergistic? -Comprehensive quantitative research based on content analysis
Source: PLoS One. 2024 Mar 7;19(3):e0298601. doi: 10.1371/journal.pone.0298601 (PMC10919601; doi:10.1371/journal.pone.0298601)
Supplement: S1 Data — (ZIP) [file pone.0298601.s001.zip › Data/地级市Z_F工作报告.zip!/╕ú╜¿/╞╬╠∩╩╨/└┤╫╘╣1⁄2╓┌║┼í╛┬φ┐╦╩2╛▌═°í┐_2015.docx]

各位代表：

　　现在，我代表莆田市人民政府向大会作政府工作报告，请予审议，并请市政协各位委员和其他列席的同志提出意见。

　　一、2014年工作回顾

　　过去的一年，在中共福建省委、省政府和中共莆田市委的坚强领导下，我市各级政府深入学习贯彻党的十八大、十八届三中、四中全会和习近平总书记系列重要讲话精神，全力抓好稳增长、调结构、促改革、惠民生等各项工作，经济社会平稳健康发展。初步统计，全市生产总值1502.1亿元、增长11.1%；财政总收入175.08亿元、增长14.3%，地方财政收入110.30亿元、增长16.2%；全社会固定资产投资1452.4亿元、增长21.9%；外贸出口33.1亿美元、增长5.0%；实际利用外商直接投资3.4亿美元、增长13.0%；社会消费品零售总额480.3亿元、增长12.1%；按新的统计口径，城镇居民人均可支配收入26871元、增长9%，农村居民人均可支配收入12829元、增长10.7%；居民消费价格总水平上涨2%；城镇登记失业2%；人口自然增长率6.6‰；完成节能减排年度任务。受宏观环境、价格因素等影响，全市财政总收入和生产总值与预期有差距，但增幅分别居全省第一位和第二位。

　　一年来的主要工作及成效是：

　　（一）全力以赴稳增长。面对严峻复杂的经济形势，快速应对处置，及时出台工业转型升级、支持龙头企业、扶持小微企业、稳定外贸出口、企业融资服务、促进港口发展等一系列政策措施，增强企业发展信心。强化金融服务实体经济，新增银行金融机构9家、海交所挂牌企业6家，金融机构本外币存款余额1450.2亿元、增长12%，贷款余额1331.4亿元、增长21.6%。建立小微出口企业“信保易”服务平台，开展“万家小微企业成长贷”服务，小微企业贷款余额202.8亿元、增长19.1%；设立电子商务、海洋牧场、木材加工、家庭旅馆等互助成长资金，推进工业厂房历史遗留办证，缓解企业融资难、融资贵问题；成立政府性融资再担保公司，设立应急保障周转金，实现转贷57.9亿元、惠及137家企业；打击“逃废债”行为，全力做好“控新化旧”工作，金融形势总体平稳。搭建艺博会、红博会、啤酒节等平台，组织产销联盟，促进本地产品销售。完成我市第三次全国经济普查任务，获得全国先进集体称号。

　　（二）三次产业协调发展。突围临港产业、文化产业、设施农业、海洋牧场、电子商务、医疗产业等取得初步成效，三次产业比重由2013年的8.5:58.4:33.1调整为7.3:57.7:35。

　　第一产业增加值109.8亿元，增长3.4%。依托“一区两园”，推广利农模式，促进土地流转，新建大棚果蔬8000亩；推动畜禽楼房养殖，新增生猪存栏3.4万头、家禽80万羽；促进林下经济、花卉苗圃基地多样化发展；发展远洋捕捞，改造大型渔船50艘；157家市级以上农业产业化龙头企业实现销售收入180亿元。

　　第二产业增加值866.8亿元，增长11.9%。新增规模以上工业企业120家、总数达1037家，规模以上工业增加值696.2亿元，增长13.0%。新增资质建筑企业35家、总数达238家，建筑业增加值165.6亿元，增长12.5%。实施产业龙头促进计划，福建LNG站线公司产值突破百亿元，差别化纤维等项目建成投产，PA6等项目加快建设，CPL等项目前期取得突破。推进科技创新型城市试点工作，获得专利授权2175项，增长27.6%；规模以上高技术产业产值170.4亿元，增长5.3%；技改投资212.8亿元，新增产值134亿元。

　　第三产业增加值525.5亿元，增长11.4%，增幅居全省第一位。新增第三产业市场主体2.5万家，增长39.2%；新增限额以上商贸企业203家、总数达641家。文化产业增加值131亿元，增长11.8%。新增限上电商企业69家、总数达100家，零售网商密度、网商发展指数分别位列全国百强城市第5位、第18位。全年接待游客1725.1万人次、增长18.1%，旅游总收入136.2亿元、增长19.5%。获得“中华美食名城”称号。

　　（三）项目建设取得实效。460个市重点项目完成投资1092亿元，其中，“双百”开竣工项目实现开工97个、竣工97个；投资超亿元项目248个、投资超10亿元项目20个；对接央企、民企、外企项目300个，完成投资746亿元；与省属企业对接合作签约项目48个，总投资959亿元。城乡基础设施提升工程扎实推进，建成国投湄洲湾煤炭码头一期工程、向莆铁路莆田货运东站、福厦铁路仙游客运站等项目；推进湄洲湾港口铁路国投支线、湄渝高速公路莆田段等项目，铁路运营总里程达134公里、高速公路通车总里程达184公里、普通国省干线通车总里程达236公里、电力装机容量达431万千瓦，港口吞吐能力建成3770万吨、在建4714万吨，具备300万方以上木材装卸能力。开展东圳水库水环境综合治理，木兰溪防洪工程、乌溪水库等加快建设，金钟水利枢纽及引水配套工程主城区全线贯通。

　　（四）新型城镇化统筹推进。着力解决人进城、建好城、管好城问题，城镇化率达55.3%。颁布实施《莆田市城乡一体化总体规划》，推进“四规合一”。深化户籍制度改革，迁移户口9619人次，农业转移人口市民化有序推进。实施宜居环境建设行动计划，深化“点线面”攻坚，整治城市“三边三节点”项目39个，整治村庄166个，创建美丽乡村96个，打造特色景观带8条。推进市政设施建设，新改扩建雨水管网124公里、污水管网230公里、供水管网80公里、燃气管网105公里、城市道路155公里、绿道60公里。推进城市停车场、透水率、片林、湿地及绿色建筑“五项行动”，新增停车位4046个、绿地181公顷。综合治理城区内河45公里，除险加固中小型水库21座，新保障27万群众饮水安全。新建、改造公交停靠站82个，新投放公交车216台。规范城乡建设用地秩序，拆除“两违”建筑217万平方米，清理闲置土地1408亩。加强生态保护，推进节能减排，防治水、大气、土壤污染，拆除关闭畜禽养殖场5629家，4个县区通过省级生态县（区）考核验收，空气质量连续四年位居全省第一位。4个省级小城镇实施项目164个，完成投资136亿元；“幸福家园”3个试点镇和46个试点村实施项目313个，完成投资27亿元；榜头镇列入首批省级小城市试点。扶持50个老少边岛扶贫开发重点村，兑现补助资金1.3亿元。

　　（五）改革开放继续深化。城乡一体化综合配套改革、政府职能转变和机构改革、要素资源市场化配置、社会治理创新等四大领域34项年度重点改革任务扎实推进。新型城镇化、民营经济综合改革、电子商务、新能源、信息惠民等列入国家改革试点。公布市级行政审批目录清单，建立标准化服务体系，投用市行政服务中心大楼，取消43项、下放152项市级审批和服务事项，审批效率提高60%以上。推进政府采购改革，引入电商平台作为供应商。在全省率先组建食品药品监管机构，有序推进工商、质监等机构改革，建成市场监督管理信息平台。 （上接第1版）实施工商登记制度改革，新登记企业数量和注册资本分别增长45%、40%。建立企业征信机制，34家企业被认定为省级工商优异企业，数量居全省第三位。组建旅游集团、南日集团，整合市属国有企业，资产总额达420亿元。壮大重点出口行业，培育出口品牌，拓展口岸货源腹地，设立首家天喔食品保税仓库，稳定外贸增长。口岸外贸吞吐量达1345万吨，新增莆头1#码头外贸作业点。进口木材163万方，带动木材加工产值47.2亿元。组织参加“9·8”投洽会、赴东南亚招商等活动，签约外资项目41个、总投资27亿美元。新批台资项目4个，莆台联创歌曲《同圆中国梦》在央视跨年元旦晚会播出，六个乡镇与台湾建立对口联系。加强侨务工作，发挥侨力、引进侨智、涵养侨源、服务侨胞成效明显。新增澳大利亚帕拉玛塔市、斯里兰卡奇洛市两个国际友好城市。闽东北经济协作、福莆宁岚同城化继续推进。

　　（六）公共事业全面发展。市财政投入民生社会事业支出118亿元，占财政支出74.3%。16件为民办实事项目完成年度任务。新增中小学学位1.1万个、幼儿园学位3200个，75%以上中小学班级配备“班班通”多媒体教学设备，推进中小学招生制度改革，新增4个国家级“义务教育发展基本均衡县”。推进医改工作，新增医疗床位800张，实行药品零差率销售，放开二级甲等及以上医院医师多点执业，实施市、县两级医院挂钩帮扶乡镇卫生院，成立莆田（中国）健康产业总会，列入省社会资本办医试点城市。实施文化走出去工程，组织“海上丝绸之路”油画艺术周等活动，4位莆籍画家作品入选第十二届全国美术作品展，莆田再次荣获中国摄影之乡、中国摄影创作基地称号。“三馆一宫”、社区电子阅览室、数字农家书屋、文化激情广场等文化惠民工程加快建设，组织各类公益演出160多场次，举办道德讲坛3000多场次，加演莆仙戏现代文明小戏1000多场次，通过第12届省级文明城市考评。木兰陂列入世界首批灌溉工程遗产名录，5个项目入选第四批国家级“非遗”名单。成功举办市全民健身运动会，承办国际男子网球巡回赛、全国射击总决赛、射箭冠军赛等。成功防控登革热等疫情，卫生应急处置能力不断增强。加强计划生育工作，实施“单独两孩”政策。保密、档案、气象、地震、贸促会、地方志、民族宗教、红十字会等工作取得新进步。

　　（七）社会保障不断增强。实施积极就业政策，新增城镇就业2.3万人，农民工劳动合同签约率达96%，劳动争议受理案件结案率达97.6%。实现居民养老保险和医疗保险城乡统筹并轨，城乡居民基础养老金标准提高到每月70元，将失独家庭、村主干等纳入城镇职工养老保险。城乡居民基本医疗保险筹资水平提高到每人每年390元，实施城乡医疗救助7.9万人次。城市、农村低保标准分别提高到每人每月468元、350元，发放城乡低保金、五保金2.3亿元，惠及9.6万困难群众。有效调控房地产市场，推行“公转商”等政策，发放公积金贷款10.3亿元。安置房新开工项目18个、面积126万平方米，竣工项目35个、面积178万平方米，新回迁1982户、办证21266套；新开工保障性住房1013套；造福工程搬迁2920户、13364人。推进数字莆田建设，建立电子政务公共平台、智慧城市公共信息平台，拓展城市一卡通应用领域。成功防御台风、暴雨袭击，安全转移群众6.9万人次。推进平安莆田建设，开展“六五”普法，建设城乡社区网格化系统，刑事案件立案量下降9%，安全生产形势总体稳定。建立“两挂钩一督导”信访责任落实机制，有效化解初信初访和信访积案。加强双拥工作，提高优抚安置标准，健全军民融合发展机制。

　　（八）自身建设持续加强。深入开展党的群众路线教育实践活动，坚持“四下基层”，纠正“四风”问题，着重解决好联系服务群众“最后一公里”问题。严格落实中央八项规定、国务院“约法三章”，停止新建楼堂馆所，完成办公用房清理，强化机构编制管理，全市“三公”经费支出下降31%，以市政府名义召开的会议减少12%、下发文件减少18%。坚持依法行政，主动接受人大、政协和社会舆论监督，认真办理市人大代表建议168件、市政协提案205件，办结率均为100%。开展党风政风人民评电视直播活动，行政监察和审计监督力度加大，廉政建设和反腐败工作扎实推进。

　　过去的一年，我们遇到的挑战比预想的要多，成绩的取得来之不易，得益于中共福建省委、省政府和中共莆田市委的正确领导，得益于全市人民齐心合力、团结奋进和各方面的支持。在此，我代表市人民政府，向全市人民，向人大代表、政协委员、各民主党派、工商联、各人民团体、无党派人士、离退休老同志和社会各界人士，向中央、省直各部门及驻莆机构、驻莆部队、武警官兵、公安民警，向关心支持莆田发展的港澳同胞、台湾同胞、海外侨胞和国际友人，表示衷心的感谢！

　　当前，我市经济社会发展中还存在不少困难和问题，主要表现在：一是临港产业优势还未充分发挥，主导产业不明显，龙头企业不多，规模企业偏少，重大产业项目新投产少。二是部分企业生产经营困难，融资难、融资贵问题还没有根本解决。三是重大项目前期工作不扎实，土地征迁难、项目推进慢。四是优质教育、医疗等资源总量不足、分布不均。五是城市建设管理和环境保护还要进一步加强，食品药品、安全生产、社会治安等领域还存在不少隐患。六是改革创新力度还不够大，政府职能转变还不到位，法治能力有待提升，作风建设有待加强。对此，我们要认真对待，采取更加有力的措施加以解决。

　　二、2015年工作安排

　　做好今年工作，必须认清国内外经济形势，把思想和行动统一到中央和省委、省政府的分析判断上来，既要看到发展阶段转换带来的新挑战，更要看到新常态孕育的新机遇，认识新常态、适应新常态、引领新常态，主动作为、顺势而为，确保“十二五”圆满收官，科学编制“十三五”规划，加快推进莆田科学发展跨越发展。

　　政府工作的总体要求是：全面贯彻党的十八大、十八届三中、四中全会、中央经济工作会议、省委九届十二次全会、全省经济工作会议和市委六届八次全会精神，深入学习贯彻习近平总书记系列重要讲话精神，认真落实中央支持福建进一步加快经济社会发展的一系列政策措施，按照建设机制活、产业优、百姓富、生态美新福建的要求，主动适应我国经济发展新常态，坚持稳中求进的工作总基调，以提高经济发展的质量和效益为中心，以港兴市、产业强市，把发展临港先进制造业作为战略支撑，壮大实体经济，培育产业龙头，突出创新驱动，统筹城乡发展，深化改革开放，加强民生保障，建设生态文明，坚持好中求快，努力打造宜业、宜商、宜居的莆田。

　　今年经济社会发展的主要预期目标是：地区生产总值增长11.5%左右、预期1700亿元；财政总收入增长14%左右、预期200亿元，其中，地方财政收入增长11%左右、预期122亿元；全社会固定资产投资增长23%左右、预期1780亿元；社会消费品零售总额增长14%、预期548亿元；外贸出口增长7%、预期35.4亿美元；实际利用外商直接投资增长10%、预期3.75亿美元；城镇居民人均可支配收入增长9%；农村居民人均可支配收入增长11%；居民消费价格涨幅控制在3%左右；城镇登记失业率控制在3%以内；人口自然增长率控制在9‰以内；完成年度节能减排任务。

　　重点抓好以下六个方面工作：

　　（一）深入挖掘潜力，扩大三大需求

　　强化投资支撑作用。实施科学发展跨越发展行动计划，落实市长一月一调度、副市长两周一协调机制。推进500个市重点项目建设，完成年度投资980亿元，落实县区（管委会）属地项目负责制，确保“双百”开竣工项目限时间开工、促提前竣工、按节点突破。加强项目策划储备和动态管理，突出招商选资，深化“三维”项目对接。推行建设工程质量安全标准化管理，打造阳光工程、优质工程。

　　拓展城乡基础设施提升工程。开工建设莆永高速湄洲连接线、涵江港至江阴港跨海大桥等项目，加快建设罗屿40万吨铁矿石码头、湄洲湾国投煤炭码头二期工程、联十一线等项目，建成湄洲湾港口铁路罗屿支线、国投煤炭码头支线、湄渝高速公路莆田段、宁海新桥等项目。优化市政路网结构，加快建设木兰大道、壶公路、白塘路等项目，改造瓶颈路段，提升一批主街道，促进道路交通更加便捷通畅。统筹防洪安全、水环境治理、景观建设，推进木兰溪防洪工程。加快建设乌溪水库、西音水库、市第三水厂、妈祖城水厂及配套管网等，建成金钟引水配套工程。加快推进LNG5#6#储罐、南日岛和平海湾海上风电、国投湄洲湾第二发电厂等项目。实施信息惠民工程，建设智慧城市支撑平台和运行管控中心。

　　发挥投资对产业结构调整的引领作用。鼓励引导先进产能扩大生产规模，壮大总量、提升存量，推动规模以上工业产值净增300亿元以上，工业增加值净增100亿元以上。重点跟踪滨海化工等100个新增长点企业，争取新增规模工业企业100家以上、产值超20亿元企业10家以上；挖掘技改投资潜力，推进华峰新材料等100个重点技改项目，带动技改投资280亿元以上，新增产值130亿元以上。

　　有效促进房地产投资和销售。落实房地产调控政策，把握供地重点、节奏、布局、投向，增加中小套型普通商品住房供应，推动土地、房产二次抵押登记，简化规划审批、验收手续，加快开发玉湖新城、妈祖城等项目。发挥好公积金的调节作用，扩大首套房、首改房需求，激活二手房和住房租赁市场，促进房地产市场健康发展。

　　推进重点领域消费。实施信息、绿色、住房、旅游休闲、教育文体、养老健康家政等六大消费工程，健全消费维权和社会信用体系。支持网购发展和农村电商配送，培育消费新业态。发挥湄洲岛龙头带动作用，主推“妈祖故乡、工艺莆田”品牌，实施旅游十大提升工程，发展休闲农业和生态观光农业，建设“森林人家”，打造“滨海朝圣、禅修习武、文化体验、休闲养生”四大旅游经济带。提升工艺美术城、仙游博览城、上塘珠宝城等经营管理水平，促进文化创意和设计服务与其他相关产业融合发展，推动高端消费向大众消费转型。加强农产品质量安全监管，扶持无公害农产品、绿色食品、有机食品和保健品等生产企业，发展护理、养老等健康服务业。持续开展本市企业“手拉手”活动，推广啤酒节、美食节等做法，鼓励大型商场、超市、加油站商店等采购本地产品。

　　促进外贸稳定增长。积极主动对接和借鉴福建自贸区政策，建立投资准入、贸易便利化等新机制。落实外贸稳增长政策，实行“一对一”重点出口企业扶持制度，注重品牌培育，优化退税流程，改善贸易环境，形成政策叠加效应，促进货源回流。用好“助保贷”、“信保易”政策，发挥中国鞋业出口基地作用，创建果蔬、水产品、油画等出口基地，增强出口产品竞争优势。鼓励先进技术装备和资源型产品进口，做大木材进口贸易。

　　积极融入“一带一路”建设。加强与沿线城市合作，共同推进“海上丝绸之路”申遗工作，促进基础设施互联互通、经贸合作、人文交流等，着力打造“海上丝绸之路”核心区。借力妈祖文化，发挥侨的优势，做好新华侨、侨二代、侨三代等侨情普查，加强双向交流合作，精准招商推介，鼓励侨商卖莆货，探索政府引导、企业对接、产业升级的外经贸模式。

　　促进莆台融合发展。建设湄洲岛第二通道及对台客滚码头，申报赴台“个人游”试点，推进两岸直航常态化。办好湄洲妈祖文化旅游节、海峡论坛·妈祖文化活动周、妈祖春秋祭典等活动，增进两岸本源认同。做强做优台湾农民创业园，建设湄洲岛妈祖文化创意产业园，打造湄洲湾莆台产业对接集中区。

　　（二）优化产业布局，推动产业集聚

　　加快工业转型升级。健全以技术创新、生态环保、税收贡献为导向的产业发展促进机制，兑现各项扶持政策，做好政策储备。落实好金融扶持实体经济发展措施，充分发挥“一个行业、一个政策、一个协会、一个资金池”机制作用，扶持小微企业健康发展，通过“增信增贷”，解决有市场、有订单、有效益企业的资金困难；通过“高位嫁接”，支持优势企业通过合资合作、资产收购和股权置换等方式，兼并重组困难企业；通过“控新化旧”，分类处置不良贷款，防止新的不良传导，稳住工业基本面。注重创新驱动，实施引智兴莆和“十大科技创新工程”，提升莆田高新技术园区，建好中科院海西研究院莆田中心，争创全国科技进步先进城市“六连冠”。落实研发费用加计扣除等普惠性政策，加快“机器换人”、“电商换市”，鼓励商业模式创新，促进鞋服等传统产业转型升级。

　　大力发展临港先进制造业。实施产业龙头促进计划，着力发展化工新材料、食品加工、工艺美术三个千亿支柱产业，培育发展高端装备、医疗健康、电子信息三个500亿新兴产业。瞄准行业龙头企业、产业链核心环节和缺失项目，引进、培育一批体量大、关联度高、产业链长的龙头项目，引导中小企业围绕龙头开展协作配套，促进产业集聚发展。化工新材料产业重点发展CPL、PA6等，带动拉丝、纺织等产业发展；食品加工产业重点发展啤酒、农产品、海产品、粮油精深加工等，带动纸箱、冠盖、物流、仓储等产业发展；工艺美术产业重点发展家具、雕刻、油画、珠宝首饰等，打造一批产业集聚基地；高端装备产业重点发展数控机床、智能装备、新能源汽车等，建设激光精密机械产业园，打造涵江装备制造产业集中区；医疗健康产业重点发展高端耗材、医疗器械、养老康复，配套建设高端专科医院、医疗营销总部、耗材设备交易中心和研发生产基地；电子信息产业重点发展集成电路、高世代面板等。

　　优化工业园区产业布局。理顺工业园区管理体制，完善配套设施及服务体系。盘活存量、做优增量，清理整合闲置土地、可开发利用土地，实行差别化工业用地管理，提高土地利用效益。按照一个园区一至两个主导产业的要求，实行差异化发展，石门澳工业园重点发展化工新材料产业；东吴临港工业园重点发展临港制造业、食品加工业；兴化湾南岸工业园重点发展高端装备、电子信息产业；莆头工业园重点发展海洋重工、建筑机械等大型成套设备产业；仙港工业园重点发展先进制造业，承接城区产业外移；东峤工业园重点发展纺织产业。

　　提升第三产业水平。促进服务业大发展，推进生产性服务业与实体经济在更高水平上有机融合，促进生活性服务业提质扩面。鼓励更多金融机构入驻莆田，支持扩大规模、拓展业务、创新产品，争取新设一批村镇银行，发展一批财务公司、融资性担保机构、小额贷款和租赁公司，支持互联网金融规范发展。推进火车站站前物流园、莆头木材集散基地、黄石商贸物流园等项目，新建一批保税仓，完善仓储、冷链物流设施，促进外挂车辆回归，培育、引进一批物流龙头企业入驻本地物流中心。围绕提升莆田网军、打造电商名城，加强示范基地建设，建设垂直细分行业电商平台，推进跨境贸易电子商务试点，争取设立一般贸易“跨境零售海关监管中心”。推进60个重点商贸项目，实现投资150亿元；新增限上商贸企业180家，实现商品销售额1400亿元。依托总部商务区，加大招商力度，促进莆商回归，培育楼宇经济。积极发展投资管理、会展、会计、设计、评估、咨询、律师等现代服务业。

　　发展特色现代农业。落实强农惠农富农政策，构建农业新型经营体系，引导园艺、畜牧、水产、林竹等向全产业链拓展，以二产加工现代化带动一产规模化。严守基本农田红线，建设一批高标准农田，保障粮食安全。提升“一区两园”建设，实施福建精致农业产业园等40个设施农业项目，新投产设施果蔬6千亩，楼房养猪新增出栏4万头，大棚养禽新增100万羽。推广应用农业科技，实施种业创新和产业化工程，扶持发展林下经济、花卉苗木，促进多样化发展。保护和振兴四大名果，提升甜柿、牛樟等种植效益，每个县（区）培育3个以上县级家庭农场示范点。引导和规范土地经营权有序流转，支持适度规模经营，培育壮大农业龙头企业。加强农村金融服务，建立农业资金池，探索设立副食品价格调节基金。

　　发展现代渔业。加快建设闽台渔业合作产业园、后海现代渔业产业园、对虾养殖加工产业基地等，促进涉海企业、项目向园区集聚。立体开发南日岛海上风电与海洋牧场，加快渔港建设，拓展养殖空间，建成15个国家级水产健康养殖示范场。坚持扩大生产与精细加工并重，提高鲍鱼、花蛤、海带、海参、东方螺、石斑鱼等主导水产品附加值，打响南日鲍、莆田花蛤等品牌。

　　（三）坚持城乡一体，建设新型城镇

　　有序推进农业转移人口市民化。落实莆田市国家新型城镇化综合试点工作方案。推行无门槛居住证制度和差别化落户政策，加强农业转移人口技能培训，提升进城人员就业创业能力。增强产业支撑，培育特色鲜明、竞争力强的优势产业，发展县域经济，吸纳转移人口就业。解决教育、医疗、住房等现实需求，推进就业服务、公共教育、基础养老、基本医疗等覆盖城镇常住人口。

　　优化城乡布局。实施《莆田市城乡一体化总体规划》，抓好重点专项规划修编，完成第二批、第三批试点镇村规划编制，确保一张蓝图绘到底。推动“一溪两岸”联动发展、城涵同城化发展，做大做美仙游城关，加快发展滨海新区。推进榜头镇省级小城市试点建设，支持埭头、枫亭、度尾等中心镇与周边乡镇连片融合发展。实施幸福家园“十镇百村”工程，推广土地流转、确权发证、环境治理、新型社区建设等经验做法，推进城乡规划、基础设施、产业发展、公共服务、社会治理一体化。注重科学扶贫和精准扶贫，引导资源要素向50个老少边岛扶贫开发重点村倾斜，培育“一村一品一业”，增强造血功能。

　　抓好宜居城乡建设。延伸拓展“点线面”攻坚，提升城市“三边三节点”整治水平，继续推进城区内河、城涵河道综合整治，促进绿道网连线成片、互联互通，打造一批美丽乡村景观带。创建国家森林城市，提升“六山”公园和土海湿地公园，加强铁路、高速公路、市政道路、河道等两侧绿化和管护，把森林引入城市，把花园建在街区。加大城中村、城市棚户区、城乡结合部、低洼易涝片区改造力度，实施龙德井、塘北等片区改造项目，强化市政设施配套和建设管理。提升公交和出租车服务水平，新增公交车120辆、出租汽车100辆，推进城乡公交客运一体化。深入开展道路交通整治，加强市容市貌、背街小巷、地下空间、流动人口和夜市的动态管理。保持打击“两违”高压态势，优化拆后土地利用，完善农村建房审批。

　　（四）突出生态示范，促进环境友好

　　完善环保设施。建设污水管网，推进雨污分流，确保排污大户接入管网，完成乡镇污水处理设施及管网建设。推进环湄洲湾北岸尾水排放管道工程、市垃圾焚烧发电厂三期扩建等，建成闽中污水处理厂三期扩建、仙游第二污水处理厂。加强污水处理厂运行监管，确保达标排放。完善垃圾收运系统，推行垃圾分类收集、循环利用。

　　狠抓节能减排。坚守环保底线，严把项目节能环保关，发展绿色经济、循环经济、低碳经济。严格落实环保监管“一岗双责”和分级管控制度，健全企业环保失信追责和环境损害赔偿制度。开展中心城区、工业园区、主要集镇及木兰溪、萩芦溪两岸排污口整治，严厉打击违法排污行为。加快循环经济示范企业、园区建设，推行清洁生产。加快淘汰黄标车、老旧汽车，推进重点产业园区集中供热、“煤改气”、“煤改电”等清洁化改造，强化脱硫设施运行管理，治理建筑工地扬尘、噪声、餐饮油烟等污染，确保空气质量持续位居全省前列。

　　加强生态保护。创建全国环保模范城市、国家级生态市、水生态文明建设试点城市和生态文明先行示范区，完善生态补偿和资源有偿使用制度，保护好城市“绿心”。完成生态功能红线划定，加强水资源“三条红线”管理，加快东圳水库、外度水库饮用水源一级保护区居民点搬迁，建设环库生态防污带。深化村庄整治、畜禽养殖污染整治，健全农村保洁长效机制。加强环境监测，打击破坏生态环境违法行为，推进一重山、海岛等造林绿化和生态修复，打造青山绿水、碧海蓝天的美丽莆田。

　　（五）继续深化改革，释放发展红利

　　深化行政体制改革。加快政府机构改革和体制调整，推进事业单位分类改革。扩大政府购买服务试点。优化行政服务中心功能，推行行政审批标准化、规范化和网上审批，再取消和下放一批市级行政审批事项，坚持放管结合，强化事中事后监管，切实提高审批效率，优化发展环境。加快港口管理体制改革，更好地服务我市港口建设。

　　激发国有企业活力。理顺国有资产监管体制，健全企业负责人业绩考核和薪酬管理制度，加强财务和经济责任审计，完善现代企业管理制度。做大做强主业、优化配置资源，完成党政机关与所属企业及经营性资产脱钩改制，进一步盘活国有资产存量，更好地发挥国有企业生力军作用。

　　创新投融资体制机制。落实“非禁即入”政策，推广政府和社会资本合作模式，引导和支持民间资本进入基础产业、基础设施、社会事业等领域。整合部门专项资金，通过设立投资基金、担保基金等市场化模式，激发社会资本投资潜力。防范金融风险，打击非法集资、高利贷行为，改善金融环境。规范土地招拍挂和用地管理，建立统一规范的公共资源交易平台。加强农村土地承包经营权、集体建设用地使用权、宅基地使用权、农村房屋所有权、林权等确权登记发证，推进流转、抵押等机制建设。促进海洋资源市场化配置，深化海域、海岛收储试点。

　　（六）发展公共事业，改善民生福祉

　　提高公共服务供给能力。实施义务教育“全面改薄”工程，抓好城区中小学和幼儿园扩容建设，新增学位8000个，提高优质教育资源覆盖面。推进莆田学院、湄洲湾职业技术学院新扩建项目。深化公立医院改革，加强市级医疗集团管理，做好社会资本办医试点，新增医疗床位800张。完善县区、乡镇医疗卫生设施，加强村（居）卫生所建设，推动优质医疗资源向基层延伸。建成“三馆一宫”，提升公共文化服务均等化、数字化、标准化水平，繁荣发展社会科学、新闻出版、广播影视、文学艺术事业。做好文化遗产保护和传承，打造一批历史文化名镇、名街、名村和传统村落。完善城乡公共体育设施，广泛开展全民健身活动，筹备第25届世界技巧锦标赛、首届全国青年运动会射击、射箭等赛事。强化计划生育管理和服务，稳定低生育水平。

　　保障和改善民生。办好东圳水库水环境综合治理等15个为民办实事项目，织好基本民生安全保障网。落实就业创业扶持政策，引导大众创业、万众创新，加强职业教育培训，鼓励名师带徒，开展技能竞赛，培养更多产业发展需要的技能型人才，新增城镇就业2万人。坚决防止拖欠农民工工资，依法维护企业和劳动者合法权益。坚持保住底线、严守中线，进一步完善社会保障体系。健全城乡居民养老保险长缴多得保障机制，推动进城务工人员、被征地、征海农民纳入城镇社保体系。实施城乡医保大病统筹，提高医保报销水平。推进国家级工伤预防试点。加强社会救助和临时救助，实现城乡最低生活保障标准和补助水平一体化，兜住困难群众基本生活底线。持续治理餐桌污染，建设食品放心工程，确保人民群众“舌尖上的安全”。鼓励社会力量参与慈善福利事业。保障性安居工程新建13500套、基本建成7000套，继续抓好安置房建设和历史遗留办证工作，造福工程再搬迁2500户。

　　加强和创新社会治理。深化平安莆田建设，规范城乡社区网格化服务管理，实现电子监控村居全覆盖，继续推进“千名干部进村户、排忧解难保平安”活动，完善社会矛盾纠纷大调解工作体系和社会治安立体化防控体系，健全重大事项维稳风险评估机制，防范和打击破坏重点项目建设、市场经济秩序等违法犯罪活动。按照“路线图”依法处理信访事项，推进诉访分离，把涉法涉诉信访纳入法治轨道。推进国家智慧城市、信息惠民试点示范城市建设，整合市级数据中心。加强普法教育、法律援助、社会诚信和青少年思想道德建设。落实安全生产党政同责、一岗双责，提升安全管理标准化水平。加强防汛抗旱、重大疫情防控等工作，提升应急处置能力。规范住宅小区物业管理。做好村居委会换届选举工作。支持工会、共青团、妇联等开展工作。落实民族政策，规范宗教事务管理。强化全民国防意识，提高军转安置和优抚工作水平，支持驻莆部队和武警部队训练，加强民兵、预备役部队和海防、人防建设，促进军民融合深度发展，争创全国双拥模范城“五连冠”。

　　三、全力建设法治政府

　　各位代表，做好政府工作，必须更加重视自身建设，全心全意为人民服务，尽心尽力对人民负责，全力建设法治政府和服务型政府。

　　加快职能转变。推进简政放权，亮出行政权力清单，做到“法无授权不可为”；理出政府责任清单，做到“法定职责必须为”。更加注重运用法律手段调节市场活动，更加注重发挥市场在资源配置中的决定性作用，更加注重依靠政策和服务促进经济发展，用政府权力的“减法”换取市场活力的“加法”，为各类市场主体创造公平竞争的环境。

　　推进依法行政。必须在党的领导下、在法治轨道上开展工作。自觉接受市人大及其常委会的法律监督、工作监督和政协的民主监督，认真办理人大代表建议、意见及政协委员提案；强化政府层级监督和监察、审计等专门监督；重视司法、社会和舆论监督，推进新闻发布常态化。健全重大决策终身责任倒查及追究机制。落实行政执法责任制，规范公正文明执法。推进行政复议、应诉和执法监督规范化。坚持政务公开，让权力在阳光下运行。

　　加强作风建设。拓展党的群众路线教育实践活动成果，牢记“作风建设永远在路上”，坚决纠正“四风”问题，促进作风建设常态化、长效化。守纪律讲规矩，确保政令畅通。加强效能建设，整治庸懒散拖，落实“马上就办”，认真解决乱作为、不作为、慢作为问题，破除熟人经济，杜绝吃拿卡要，开展政风行风评议，提升绩效管理水平。按照“三严三实”要求，发扬“钉钉子”精神，提高政策执行力和工作落实力，坚持在基层一线化解矛盾、解决问题、推动工作，对看准的事情、定下的目标、部署的工作，锲而不舍、久久为功，善始善终、善作善成。

　　坚持廉洁从政。严格落实党风廉政建设责任制，严格执行中央八项规定和国务院“约法三章”，健全惩治和预防腐败体系。厉行节约、反对浪费，完善机关经费管理、国内差旅、因公临时出国（境）、公务接待等制度，推进公务用车制度改革，严控“三公”经费支出。严肃查处以权谋私和腐败行为，使广大公务人员心有所畏、言有所戒、行有所止，努力实现干部清正、政府清廉、政治清明。

　　各位代表，莆田正处在新一轮加快发展的关键时期。让我们紧密团结在以习近平同志为总书记的党中央周围，在中共莆田市委的坚强领导下，紧紧依靠全市人民，朝着宜业、宜商、宜居的目标，在更高起点上推进莆田科学发展跨越发展！
